# Predictive Value of Neutrophil-Lymphocyte Ratio for Long-Term Cardiovascular Event Following Coronary Artery Bypass Grafting

**DOI:** 10.21470/1678-9741-2018-0362

**Published:** 2020

**Authors:** Orcun Gurbuz, Gencehan Kumtepe, Hakan Ozkan, Ilker Hasan Karal, Yusuf Velioglu, Abdulkadir Ercan, Ahmet Yüksel, Serdar Ener

**Affiliations:** 1 Department of Cardiovascular Surgery, Meddem Hospital, Isparta, Turkey.; 2 Department of Cardiology, Bahcesehir University Faculty of Medicine, Istanbul, Turkey.; 3 Department of Cardiovascular Surgery, Samsun Hospital for Education and Research, Samsun, Turkey.; 4 Department of Cardiovascular Surgery, Abant Izzet Baysal University Faculty of Medicine, Bolu, Turkey.; 5 Department of Cardiovascular Surgery, Ceylan International Hospital, Bursa, Turkey.; 6 Department of Cardiovascular Surgery, Medicana Bursa Hospital, Bursa, Turkey.

**Keywords:** Coronary Artery Bypass, Percutaneous Coronary Intervention, Myocardial Infarction, Lymphocytes, Stroke, Neutrophils

## Abstract

**Objective:**

To investigate the predictive value of preoperative neutrophil-lymphocyte ratio (NLR) for long-term major adverse cardiac and cerebrovascular events (MACCE), which have not yet been well described, in patients undergoing coronary artery bypass grafting (CABG).

**Methods:**

The records of 751 consecutive patients who underwent elective CABG between January 2008 and January 2010 were retrospectively enrolled and stratified according to quartiles of preoperative NLR. At 7.8-year follow-up, MACCE was considered as an endpoint.

**Results:**

Overall MACCE was 11.6% of all cases. Long-term myocardial infarction, percutaneous coronary intervention, stroke and cardiovascular mortality were found associated with the upper NLR quartile (*P*<0.001, *P*<0.001, *P*=0.005, *P*<0.001, respectively). In multivariate analysis, NLR on admission remained an independent predictor of long-term MACCE (OR 1.087, 95% CI 1.026-1.151; *P*=0.004), in all EuroSCORE risk groups (*P*<0.001; *P*<0.001; *P*=0.029). The receiver operating characteristic (ROC) curve analyses revealed an NLR cut-off value of 4.32 predicting MACCE.

**Conclusion:**

NLR is a useful and readily available predictive marker of long-term MACCE following CABG, independent of the EuroSCORE.

**Table:** 

Abbreviations, acronyms & symbols				
ACT	= Activated clotting time		ICU	= Intensive care unit
CABG	= Coronary artery bypass grafting		LMCA	= Left main coronary artery
CAD	= Coronary artery disease		MACCE	= Major adverse cardiac and cerebrovascular events
COPD	= Chronic obstructive pulmonary disease		NLR	= Neutrophil-lymphocyte ratio
CPB	= Cardiopulmonary bypass		ON-BH CABG	= On-pump beating-heart CABG
CrCl	= Creatinine clearance		PCI	= Percutaneous cardiac intervention
CTnI	= Cardiac troponin I		PVD	= Peripheral vascular disease
EuroSCORE	=European System for Cardiac Operative Risk Evaluation		ROC	= Receiver operating characteristic
IABP	= Intra-aortic balloon pumping		STEMI	= ST elevation myocardial infarction

## INTRODUCTION

Chronic inflammation plays a central role in the development and progression of atherosclerosis^[1]^ and in its major complications like myocardial infarction^[2]^ and stroke^[3]^. Inflammatory biomarkers have been identified as useful predictors of clinical outcomes for coronary artery disease (CAD)^[4]^and cerebrovascular disease^[5]^. It has been shown that neutrophilia^[6]^ and relative lymphocytopenia^[7]^ are both negative prognostic indexes for outcomes in CAD. Accordingly, previous studies have demonstrated that neutrophil-lymphocyte ratio (NLR) is associated with severity of CAD^[8]^, adverse events in stable CAD^[4]^ and long-term mortality following ST-segment elevation myocardial infarction (STEMI)^[9]^. Current methods of risk assessment following coronary artery bypass grafting (CABG) have mainly focused on short-term mortality^[10,11]^. Gibson et al. showed the relationship between preoperative elevated NLR and mid-term mortality following CABG^[12]^. However, the medical literature does not contain data evaluating the association of NLR and the long-term major adverse cardiac and cerebrovascular event (MACCE) following CABG. We therefore evaluated the relations between the pre-procedural NLR and the long-term MACCE incidence in patients undergoing elective isolated CABG.

## METHODS

### Study Population

The ethical approval was obtained from the local ethics committee and the research was conducted according to the principles expressed in the Declaration of Helsinki. The population consisted of 802 consecutive patients who underwent elective isolated on-pump beating-heart CABG (ON-BH CABG) at Bursa Medical Park Hospital between January 2008 and January 2010. Exclusion criteria were as follows: critical preoperative state (need for inotropic drug support or intra-aortic balloon pumping (IABP), acute renal failure, need for respiratory support, history of preoperative cardiopulmonary resuscitation), previous heart surgery, myocardial infarction within 3 weeks (cTnI>0.01 ng/ml), or active infection and missing data. Finally, 751 patients (76.8% males and mean age 62.34±9.43) were included in this retrospective study.

### Definitions

Preoperative characteristics of the patients: age, sex, smoking status, hypertension, diabetes mellitus, hyperlipidaemia, family history of CAD, obesity (body mass index >30 kg/m^2^), chronic obstructive pulmonary disease (COPD), peripheral vascular disease (PVD), asymptomatic carotid stenosis, history of stroke, history of myocardial infarction, unstable angina pectoris, history of percutaneous cardiac intervention (PCI), European System for Cardiac Operative Risk Evaluation (EuroSCORE), left ventricular ejection fraction, mitral insufficiency, number of vessel disease, and presence of left main coronary artery (LMCA) stenosis.

The diagnosis of diabetes mellitus was based on previous history of diabetes or fasting plasma glucose ≥126 mg/dl or haemoglobin A1c ≥6.5%. The diagnosis of dyslipidaemia was based on previous history or total cholesterol ≥200 mg/dl or LDL ≥130. Vessel disease was defined as a stenosis of >50% of major epicardial coronary arteries. Estimated creatinine clearance (CrCl) was calculated using the Cockcroft-Gault formula: CrCl (ml/min) = ([140-age] × weight [kg])/(serum creatinine [mg/dl] × 72) (× 0.85 for women) from baseline blood samples. The diagnosis of COPD was based on previous history of bronchodilator treatment or the FEV1/FVC ratio <0.70. Carotid stenosis was defined as a ≥50% narrowing of the internal carotid artery. PVD was defined as arterial disease affecting the non-carotid vasculature. The left ventricular dysfunction was defined as moderate (ejection fraction 0.30-0.49) or severe (ejection fraction <0.30).

Preoperative and postoperative laboratory tests and outcomes were retrospectively collected from hospital records. Incomplete revascularization was defined as untreated ≥50% diameter stenosis in a major epicardial coronary arteries. Drainage was defined as the sum of the drainage in the first 24 hours. Consumed unit of blood was defined as the sum of the blood units used during hospital stay. Any inotropic support started in the perioperative period was determined as the perioperative need for inotropic support. Perioperative non-ST elevation myocardial infarction was defined as cardiac troponin I (cTnI) >5 µg/L during hospitalization without new electrocardiography change^[13]^. Perioperative STEMI was defined as cTnI >5 µg/L with new electrocardiography change or echocardiography evidence of a new regional wall motion abnormality. Postoperative renal failure was defined as an ≥100% increase in basal serum creatinine. Pulmonary complications were defined as pleural effusion, atelectasis, phrenic nerve palsy, diaphragmatic dysfunction, pneumonia, acute respiratory distress syndrome, pneumothorax or chylothorax.

Prolonged mechanical ventilation time was defined as total intubation time greater than 10 hours. Neurologic complication was defined as any new transient ischemic attack, stroke or encephalopathy occurring in the perioperative period. Early reoperation was defined as any hospitalization due to CABG related complications (such as sternal dehiscence, mediastinitis) or cardiovascular problems (such as myocardial infarction, congestive heart failure, rhythm disturbance, neurologic complications, pulmonary embolism).

Long-term follow-up was obtained through clinic visits, hospital records and phone calls. All-cause mortality (patient death reported by relatives or hospital records) and MACCE (STEMI, repeated CABG or PCI, need for dual-chamber pacemaker due to decompensated heart failure, stroke, cardiac related or sudden death) were determined.

### Surgical Procedure

All procedures were performed by the same surgeon or under his supervision using the ON-BH CABG technique. Following median sternotomy, left internal thoracic artery and other conduits were prepared. Heparin was administered to keep the activated clotting time (ACT) greater than 450 seconds. All procedures were performed without using aortic cross-clamping and cardioplegia. Cardiopulmonary bypass (CPB) was established with an ascending aortic arterial cannula and a right atrial two-stage venous cannula, using a membrane oxygenator and a roller pump. All patients were cooled to 32-34ºC. Mean arterial blood pressure was maintained in the range of 60-90 mmHg. Distal anastomoses were performed by end-to-side or side-to-side techniques with a running 7/0 Prolene^®^ suture, using a myocardial stabilizer device (Octopus IV, Medtronic Inc., Minneapolis, MN, US). Proximal anastomoses were performed using 6/0 Prolene^®^ suture during the heating period using an aortic side-clamp. After completion of CPB and cannula removal, heparin was neutralized with protamine providing an ACT of less than 160 seconds. Acetylsalicylic acid at a dose of 100 mg and enoxaparin 40 mg SC were initiated on the postoperative 24 hours. All patients were discharged under acetylsalicylic acid therapy.

### Laboratory Analysis and Echocardiography

Haematological indices were measured, as part of the automated complete blood count (CBC), using a Cell-Dyn 3700 haematology analyzer (Abbott Diagnostics, Santa Clara, CA, USA). Biochemical analyses were performed with the Architect ci8200 chemistry analyzer (Abbott Diagnostics, Santa Clara, CA, USA).

Transthoracic echocardiography was performed for each patient using a Vivid S3 (GE Healthcare, Milwaukee, WI, USA) with a 1.5-3.6 MHz phased array transducer.

### Clinical Endpoint

To identify the effect of the pre-procedural NLR on the long-term MACCE in patients undergoing CABG.

### Statistical Analysis

Continuous variables were expressed as mean ± standard deviation. Categorical variables are expressed as percentages. Cox regression analysis was performed to determine independent predictors of MACCE, with those variables with a *P*<0.1 in univariate analysis been included in the stepwise multivariate model. The odds ratio (OR) and 95% confidence intervals (CI) were calculated. The association between variables was tested using Spearman or Pearson correlation coefficient. Two-tailed *P*<0.05 were considered significant. Participants were classified into 4 groups using the NRL quartile values. Differences in baseline clinical characteristics among groups were examined by 1-way analysis of variance for continuous variables and chi-square (χ^2^) test for categorical variables. The cumulative survival curves for long-term MACCE were constructed using the Kaplan-Meier method, whereas differences among the NLR groups were evaluated with log-rank tests. The receiver operating characteristics (ROC) curve was used to demonstrate the sensitivity and specificity of NLR and its respective optimal cut-off value for predicting MACCE. All statistical analyses were conducted using the Statistical Package for Social Sciences (SPSS) software (version 15.0, SPSS, Chicago, Illinois, USA).

## RESULTS

The variables for which *P* was <0.05 in univariate Cox analysis (age, preoperative NLR, CrCl, EuroSCORE, hypertension, asymptomatic carotid artery stenosis, previous myocardial infarction, family history of CAD, insulin-dependent diabetes mellitus, PVD, LMCA stenosis, three-vessel disease, perioperative need for IABP or inotropic agent, length of hospital stay, prolonged respiratory period, perioperative renal failure, mean number of red blood cell transfusion units were identified as potential risk factors for MACCE – Table 1).

**Table 1:** Effects of multiple variables on MACCE in Cox regression analysis

Characteristics	Univariate analysis			Multivariate analysis		
	OR	95% CI	*P*-value	OR	95% CI	*P*-value
Demographics						
Age (years)	1.050	1.024-10.76	<0.001*			
Male sex	1.215	0.702-2.103	0.48			
Laboratory parameters						
WBC	0.997	0.918-1.083	0.99			
Neutrophil	1.120	1.026-1.222	0.011*			
Lymphocyte	0.461	0.333-0.638	<0.001*			
NLR	1.109	1.063-1.156	<0.001*	1.087	1.026-1.151	0.004*
CRP	1.045	0.974-1.122	0.21			
Hg	0.922	0.833-1.020	0.92			
CrCL	0.991	0.983-0.998	0.014*			
Risk factors and medical history						
Obesity	1.348	0.836-2.175	0.22			
EuroSCORE	1.182	1.094-1.277	<0.001*			
USAP	0.923	0.571-1.492	0.74			
History of MI (>21 days)	1.723	1.101-2.698	0.017*			
History of PCI	1.187	0.630-2.235	0.59			
Current smoker	0.795	0.501-1.261	0.32			
Family history of CAD	0.489	0.270-0.887	0.019*	0.409	0.180-0.928	0.032*
IDDM	2.280	1.205-4.313	0.011*			
Dyslipidaemia	1.092	0.667-1.785	0.72			
Hypertension	2.150	1.344-3.427	0.001*	2.575	1.416-4.683	0.002*
COPD	1.686	0.812-3.504	0.16			
Asymptomatic carotid stenosis	2.073	1.258-3.416	0.004*			
PVD	2.187	1.109-4.311	0.024*			
History of stroke	1.997	0.807-4.942	0.13			
Estimated LVEF 30-49%	1.006	0.606-1.671	0.98			
Estimated LVEF <30%	1.831	0.881-3.806	0.1			
Mild valvular insufficiency	1.378	0.827-2.298	0.21			
Angiographic findings						
LMCA stenosis	1.863	1.134-3.063	0.014*			
Average number of vessel disease	1.491	0.978-2.272	0.06			
Three-vessel disease	1.736	1.028-2.931	0.039*			
Perioperative data						
Number of distal anastomosis	1.190	0.932-1.518	0.16			
Incomplete revascularization	0.824	0.333-2.037	0.67			
Endarterectomy	1.233	0.734-2.072	0.42			
CPB time	1.012	0.988-1.036	0.33			
Perioperative IABP	7.660	2.415-24.298	0.001*			
Perioperative inotropic agent	2.962	1.705-5.147	<0.001*			
Perioperative AF	1.670	0.916-3.045	0.09			
Perioperative MI	1.098	0.505-2.387	0.81			
Prolonged respiratory period	1.520	1.137-2.032	0.005*	5.438	1.723-17.163	0.004*
Duration of ICU stay	1.008	0.999-1.017	0.08			
Pulmonary complication	1.230	0.775-1.925	0.38			
Perioperative renal failure	2.072	0.188-3.611	0.01*	3.028	1.089-2.048	0.026*
Cerebrovascular complication	2.490	1.318-4.704	0.005*			
Blood transfusion	1.281	1.137-1.442	<0.001*			
Length of hospital stay	1.353	1.206-1.518	<0.001*			

* Statistically significant difference. AF=atrial fibrillation; CAD=coronary artery disease; CI=confidence interval; COPD=chronic obstructive pulmonary disease; CPB=cardiopulmonary bypass; CrCL=creatinine clearance; CRP=C-reactive protein; EuroSCORE=European System for Cardiac Operative Risk Evaluation; Hg=haemoglobin level; IABP=intra-aortic balloon pump; ICU=intensive care unit; IDDM=insulin-dependent diabetes mellitus; LMCA=left main coronary artery; LVEF=left ventricular ejection fraction; MACCE=major cardiac and cerebrovascular event; MI=myocardial infarction; NLR=neutrophil-lymphocyte ratio; OR=odds ratio; PCI=percutaneous coronary intervention; PVD=peripheral vascular disease; USAP=unstable angina pectoris; WBC=white blood cell count

NLR was a stronger univariable predictor of MACCE (χ^2^=25.8) than the neutrophil (χ^2^=6.2) or lymphocyte count (χ^2^=14.9). Neutrophil and lymphocyte counts and the NLR are mathematically related, but when the NLR was used, its components did not provide additional prognostic information. Therefore, among these markers, only the NLR was entered in subsequent multivariate Cox analysis. In multivariate Cox regression analyses, NLR (OR 1.087, 95% CI 1.026-1.151; *P*=0.004), family history of CAD (OR 0.409, 95% CI 0.180-0.928; *P*=0.032), hypertension (OR 2.575, 95% CI 1.416-4.683; *P*=0.002), prolonged respiratory period (OR 5.438, 95% CI 1.723-17.163; *P*=0.004) and preoperative renal failure (OR 3.028, 95% CI 1.089-2.048; *P*=0.026) were still independent predictors of MACCE (Table 1). Moreover, NLR was the only predictor of MACCE in low-risk, medium-risk and high-risk EuroSCORE groups in the Cox regression analysis (OR 1.113, 95% CI 1.048-1183, *P*<0.001; OR 1.161, 95% CI 1.060-1.273, *P*<0.001; OR 1.127, 95% CI 1.012-1.255, *P*=0.029) (Table 2).    

**Table 2:** Effects of multiple variables on MACCE according to EuroSCORE Risk Score in Cox regression analysis.

	EuroSCORE ≤2			EuroSCORE 3-5			EuroSCORE ≥6		
	OR	95% CI	*P*-value	OR	95% CI	*P*-value	OR	95% CI	*P*-value
Laboratory parameters									
NLR	1.113	1.048-1.183	<0.001*	1.161	1.060-1.273	0.001*	1.127	1.012-1.255	0.029*
WBC	0.936	0.779-1.125	0.48	1.105	0.889-1.159	0.82	1.023	0.919-1.139	0.67
Lymphocyte	0.231	0.116-0.461	<0.001*	0.606	0.355-1.035	0.06	0.575	0.347-0.953	0.032*
Neutrophil	1.143	0.916-1.426	0.23	1.175	1.000-1.380	0.05	1.070	0.955-1.198	0.24
CrCL	0.993	0.979-1.007	0.99	0.989	0.975-1.003	0.11	0.993	0.982-1.005	0.27
Risk factors and medical history									
Age	1.025	0.965-1.090	0.41	1.019	0.974-1.067	0.41	1.059	1.009-1.112	0.02*
History of MI	2.016	0.740-5.494	0.17	1.426	0.666-3.057	0.36	1.213	0.616-2.387	0.57
Family history of CAD	0.635	0.234-1.722	0.37	0.594	0.240-1.469	0.26	0.236	0.57-0.984	0.047*
Hypertension	5.241	1.773-15.490	0.003*	1.899	0.877-4.115	0.1	1.532	0.772-3.042	0.22
IDDM	2.208	0.653-7.466	0.2	4.918	1.979-12.219	0.001*	0.996	0.305-3.254	0.99
Asymptomatic carotid stenosis	0.048	0-90770.430	0.68	2.408	1.088-5.325	0.03*	1.294	0.658-2.546	0.45
PVD	10.595	2.443-45.944	0.002*	1.496	0.421-5.317	0.53	1.158	0.409-3.283	0.78
Angiographic findings									
LMCA stenosis	2.427	0.948-6.211	0.065	2.211	0.974-5.021	0.05	1.200	0.545-2.641	0.65
Three-vessel disease	1.862	0.687-5.049	0.22	1.168	0.515-2.653	0.71	1.898	0.829-4.349	0.13
Perioperative data									
Perioperative IABP	-	-	-	4.871	0.661-35.918	0.12	6.150	1.463-25.854	0.013*
Perioperative inotropic agent	6.521	0.873-48.738	0.06	1.955	0.590-6.478	0.27	2.174	1.111-4.254	0.023*
Prolonged respiratory period	-	-	-	5.369	1.260-22.883	0.023*	2.010	0.477-8.467	0.34
Perioperative renal failure	0.046	0-244.968	0.48	3.861	1.800-8.282	0.001*	2.679	1.361-5.270	0.004*
Cerebrovascular complication	1.358	0.183-10.102	0.76	4.541	1.703-12.109	0.002*	1.211	0.470-3.122	0.69
Blood transfusion	1.104	0.730-1.670	0.63	1.357	1.147-1.606	<0.001*	1.094	0.899-1.331	0.37
Length of hospital stay	1.125	0.697-1.816	0.63	1.075	0.914-1.264	0.38	1.111	1.007-1.224	0.035*

* Statistically significant difference. CAD=coronary artery disease; CI=confidence interval; CrCL=creatinine clearance; EuroSCORE=European System for Cardiac Operative Risk Evaluation; IABP=intra-aortic balloon pump; IDDM=insulin-dependent diabetes mellitus; LMCA=left main coronary artery; MACCE=major cardiac and cerebrovascular event; MI=myocardial infarction; NLR=neutrophil-lymphocyte ratio; OR=odds ratio; PVD=peripheral vascular disease; WBC=white blood cell count

### Baseline Characteristics

After the evaluation of the patients’ data according to the inclusion and exclusion criteria, 751 patients were divided into four groups according to the NLR quartile. The demographic characteristics of these remaining participants are shown in Table 3 (mean duration of follow-up 78.2±15.79 months). NLR ranged from 0.17 to 27.9 (mean 3.04±2.69; interquartile range 1,4). Patients in the highest NLR quartile (quartile 4) had significantly higher C-reactive protein (*P*=0.018) and CrCl levels (*P*=0.002), more PVD (*P*=0.027), more insulin-dependent diabetes mellitus (*P*=0.018), more likely to smoke (*P*<0.001), and were also significantly older (*P*=0.003). However, the preoperative characteristics of the NLR ratio quartile groups were similar regarding EuroSCORE, sex, obesity, hypertension, hyperlipidaemia, family history of CAD, COPD, history of stroke, asymptomatic carotid stenosis, history of myocardial infarction, moderate and severe left ventricular dysfunction, unstable angina pectoris, previous PCI, LMCA stenosis and mean number of vessel disease.

**Table 3:** Baseline demographic and clinical parameters according to NLR quartiles.

Characteristics	Overall	Quartile 1	Quartile 2	Quartile 3	Quartile 4	*P*-value
	n=751	n=189	n=188	n=187	n=187	
		0.17-1.83	1.84-2.47	2.48-3.23	≥ 3.24	
Laboratory parameters						
NLR	3.04±2.69	1.3 ± 0.34	2.05 ± 0.19	2.87 ± 0.2	5.94 ± 4.07	<0.001*
WBC (×10^9^/L)	7.74±2.65	7.37±2.8	7.74±2.26	7.53±2.8	8.34±2.62	<0.001*
CRP	1.81±3.12	1.11±2.16	1.5±2.71	2.41±3.29	2.64±4.19	0.018*
Hg	11.05±2.31	10.90±2.34	11.07±2.31	11.11±2.35	11.10±2.26	0.69
CrCL	101.98±35.63	93.62±35.27	101.48±33.72	106.19±35.76	106.84±36.47	0.002*
Demographics						
Age (years)	62.34±9.43	61.41±9.52	61.52±9.72	61.88±9.42	64.57±8.76	0.003*
Male	577 (76.8)	150 (79.4)	136 (72.3)	187 (80.7)	140 (74.9)	0.18
Risk factors and medical history						
EuroSCORE	3.61±2.52	3.64±2.48	3.46±2.49	3.50±2.49	3.83±2.55	0.41
CCS	2.52±1.24	2.52±1.26	2.56±1.26	2.50±1.28	2.50±1.15	0.92
USAP	238 (31.7)	62 (32.8)	62 (33)	63 (33.7)	51 (27.3)	0.51
Obesity (BMI ≥30)	225 (33.3)	48 (25.4)	63 (33.5)	58 (31)	56 (29.9)	0.53
Current smoker	298 (39.7)	90 (47.6)	68 (36.2)	88 (47.1)	52 (27.8)	<0.001*
Hypertension	371 (49.4)	87 (46)	90 (47.9)	96 (51.3)	98 (52.4)	0.57
Dyslipidaemia	196 (26.1)	51 (27)	49 (26.1)	46 (24.6)	50 (26.7)	0.95
Family history of CAD	206 (27.4)	53 (28)	55 (29.3)	59 (31.6)	39 (20.9)	0.11
IDDM	54 (7.2)	10 (5.3)	9 (4.8)	12 (6.4)	23 (12.3)	0.018*
COPD	50 (6.7)	10 (5.3)	12 (6.4)	9 (4.8)	19 (10.2)	0.15
History of stroke	28 (3.7)	8 (4.2)	7 (3.7)	5 (2.7)	8 (4.3)	0.32
PVD	34 (4.5)	12 (6.3)	3 (1.6)	6 (3.2)	13 (7)	0.027*
Asymptomatic carotid stenosis	115 (15.3)	35 (18.5)	26 (13.8)	24 (12.8)	30 (16)	0.42
History of MI	224 (29.8)	59 (31.2)	51 (27.1)	61 (32.6)	53 (28.3)	0.63
Estimated LVEF 30-49%	191 (25.4)	46 (24.3)	51 (27.1)	53 (28.3)	41 (21.9)	0.97
Estimated LVEF <30%	48 (6.4)	13 (6.9)	9 (4.8)	9 (4.8)	17 (9.1)	0.27
Mild mitral insufficiency	79 (10.5)	19 (10.1)	17 (9)	20 (10.6)	23 (12.3)	0.62
Previous PCI	73 (9.7)	23 (12.2)	15 (8)	18 (9.6)	20 (10.7)	0.56
Angiographic findings						
Number of vessel disease	2.61±0.6	2.6±0.59	2.53±0.68	2.64±0.55	2.67±0.58	0.18
LMCA stenosis	125 (16.6)	28 (14.8)	31 (16.5)	30 (16)	36 (19.3)	0.7
Three-vessel disease	510 (67.9)	125 (66.1)	120 (63.8)	128 (68.4)	137 (73.3)	0.24

* Statistically significant difference. Values are presented as mean ± standard deviation or number (%), where appropriate. BMI=body mass index; CAD=coronary artery disease; COPD=chronic obstructive pulmonary disease; CrCL=creatinine clearance; CRP=C-reactive protein; CCS=Canadian Cardiovascular Society grading of angina; EuroSCORE=European System for Cardiac Operative Risk Evaluation; Hg=haemoglobin level; IDDM=insulin-dependent diabetes mellitus; LMCA=left main coronary artery; LVEF=left ventricular ejection fraction; MI=myocardial infarction; NLR=neutrophil-lymphocyte ratio; PCI=percutaneous coronary intervention; PVD=peripheral vascular disease; USAP=unstable angina pectoris; WBC=white blood cell count

### Perioperative and Early Postoperative Characteristics

Perioperative and early postoperative patients’ characteristics are shown in Table 4. Patients with higher NLR tended to have more prolonged mechanical ventilation time (*P*=0.038) and intensive care unit (ICU) stay (*P*=0.007). They were also more likely to require reintubation or noninvasive mechanical ventilation support (*P*=0.013). Moreover, they required more blood transfusion (*P*=0.002), more inotropic support (*P*=0.048), following CABG. Patients with higher NLR had a higher incidence of early stroke (*P*=0.034) and early mortality rate (*P*=0.011). However, operative data, postoperative cTnI levels, length of hospital stay, total blood loss, incidence of mediastinitis and 30-day MACCE were similar among NLR quartiles.

**Table 4:** Perioperative and early postoperative characteristics of the patients stratified by NLR quartiles.

Characteristics	Overall	Quartile 1	Quartile 2	Quartile 3	Quartile 4	*P*-value
	n=751	n=189	n=188	n=187	n=187	
		0.17-1.83	1.84-2.47	2.48-3.23	≥ 3.24	
Operative data						
Number of distal anastomoses	3.41±0.88	3.40±0.86	3.29±0.86	3.48±0.88	3.47±0.92	0.24
Incomplete revascularization	136 (18.1)	31 (16.4)	38 (20.2)	34 (18.2)	33 (17.6)	0.81
Number of grafted LAD	1.65±0.55	1.66±0.5	1.63±0.58	1.65±0.56	1.67±0.56	0.48
Number of grafted Cx	0.99±0.65	0.97±0.57	0.96±0.67	1±0.69	1.03±0.65	0.64
Number of grafted RCA	0.75±0.53	0.75±0.54	0.68±0.5	0.8±0.56	0.76±0.51	0.23
Endarterectomy	69 (9.2)	20 (10.6)	15 (8)	18 (9.6)	16 (8.6)	0.82
CPB time	87.83±26.5	87.45±26.68	85.92±30.57	85.1±21.55	94.52±26.47	0.76
Postoperative data						
Hospital stay (days)	5.84±2.71	5.70±1.59	5.83±2.79	5.62±1.94	6.22±3.91	0.21
Prolonged mechanical ventilation time	19 (2.5)	2 (1.1)	3 (1.6)	4 (2.1)	10 (5.3)	0.038*
Intensive care unit stay (h)	22.8±45.81	19.15±6.45	22.02±25.25	20.55±16.79	29.54±86.21	0.007*
Drainage (ml/24 h)	511.63±298.83	508.65±304.34	500±252.55	496.9±308.31	544.71±330.99	0.7
Blood transfusion (unit)	1.35±1.54	1.24±1.39	1.23±1.58	1.25±1.42	1.69±1.71	0.002*
cTnI (ng/ml)	2.48±2.99	2.02±2.13	2.33±2.92	2.6±3.52	2.89±3.16	0.09
Perioperative inotropic support	42 (5.6)	7 (3.7)	9 (4.8)	8 (4.3)	18 (9.6)	0.048*
Perioperative IABP	7 (0.9)	1 (0.5)	2 (1.1)	1 (0.5)	3 (1.6)	0.65
Perioperative NSTEMI	62 (8.3)	20 (10.6)	14 (7.4)	15 (8)	16 (8.6)	0.72
Early STEMI (<30 days)	6	1 (0.5)	0	1 (0.5)	4 (2.1)	0.1
Perioperative AF	94 (12.5)	16 (8.5)	27 (14.4)	24 (12.8)	27 (14.4)	0.25
Perioperative renal failure	103 (13.7)	25 (13.2)	20 (10.6)	29 (15.5)	29 (15.5)	0.46
Pulmonary complication	21 (2.8)	4 (2.1)	6 (3.1)	3 (1.6)	8 (4.3)	0.4
Reintubation/NIMV	11 (1.4)	1 (0.5)	3 (1.6)	0	7 (3.7)	0.013*
Neurological complication	52 (6.9)	14 (7.4)	9 (8.4)	13 (7)	16 (8.4)	0.53
Encephalopathy	22 (2.9)	4 (2.1)	6 (3.2)	4 (2.1)	8 (4.3)	0.55
TIA	28 (3.7)	11 (5.8)	3 (1.6)	8 (4.3)	6 (3.2)	0.17
Stroke	5 (0.7)	0	0	1 (0.5)	4 (2.1)	0.034*
Mediastinitis	5 (0.7)	1 (0.5)	0	2 (1.1)	2 (1.1)	0.16
Early reoperation	12 (2.3)	4 (2.1)	3 (1.6)	2 (1.1)	3 (1.6)	0.49
Early reoperation due to bleeding	10 (1.3)	3 (1.6)	3 (1.6)	2 (1.1)	2 (1.1)	0.94
Early rehospitalisation (<30 days)	48 (6.4)	15 (7.9)	6 (3.2)	14 (7.5)	13 (7)	0.06
Mortality (<30 days)	8 (1.1)	1 (0.5)	1 (0.5)	0	6 (3.2)	0.011*
MACCE (<30 days)	19 (2.5)	4 (2.1)	2 (1.1)	4 (2.1)	9 (4.8)	0.12

* Statistically significant difference. Values are presented as mean ± standard deviation or number (%), where appropriate. AF=atrial fibrillation; CPB=cardiopulmonary bypass; Cx=circumflex coronary artery; cTnI=cardiac troponin I; FFP=fresh frozen plasma; IABP=intra-aortic balloon pump; LAD=left anterior descending coronary artery; LIMA=left internal mammary artery; MACCE=major adverse cardiac and cerebrovascular event; MI=myocardial infarction; NIMV=non-invasive mechanical ventilation; NSTEMI=non-ST elevation myocardial infarction; RCA=right coronary artery; STEMI= ST elevation myocardial infarction; TIA=transient ischemic attack

### Long-Term Follow-Up

**Fig. 1:**
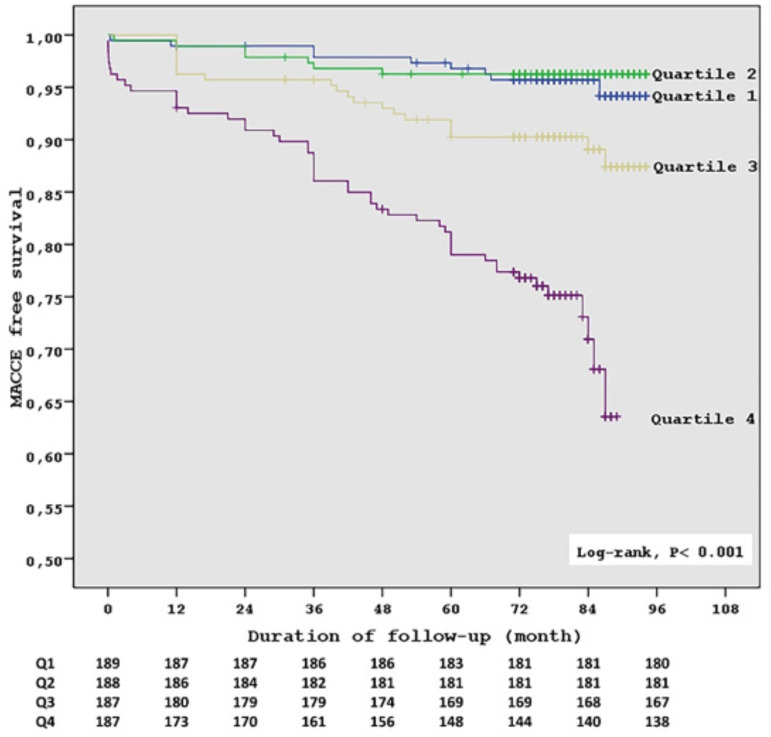
Kaplan-Meier estimates of survival free of cardiovascular death, stroke, myocardial infarction and repeated coronary revascularization (P<0.001 by the log-rank test). Q=quartile

Long-term follow-up characteristics of patients are shown in Table 5. By 7.8 years, 87 (11.6%) of the patients presented MACCE. Patients with higher NLR had lower MACCE-free survival (*P*<0.001). Accordingly, the cumulative MACCE at one year (*P*=0.001), at three years (*P*<0.001) and at 7.8 years (*P*<0.001) detected significantly more in the upper quartile group. Moreover, patients with higher NLR had higher cardiovascular mortality (*P*<0.001), more STEMI (*P*<0.001), more stroke (*P*=0.005), and more PCI (*P*<0.001). However, higher NLR was not related to non-cardiovascular mortality. Kaplan-Meier survival analysis of freedom from MACCE revealed significantly lower event-free survival in the upper NLR quartile (P<0.001 by log-rank test) (Figure 1).

**Table 5:** Long-term outcomes of the patients, according to NLR quartiles.

Characteristics	Overall	Quartile 1	Quartile 2	Quartile 3	Quartile 4	*P*-value
	n=751	n=189	n=188	n=187	n=187	
		0.17-1.83	1.84-2.47	2.48-3.23	≥3.24	
Mean follow-up time (months)	78.2±15.79	80.83±12.13	80.3±12.13	79.37±14.36	72.25±20.65	<0.001*
MACCE-free survival	76.03±18.35	80.03±13.41	79.83±13.81	76.66±18.33	67.32±23.33	<0.001*
MACCE (1 year)	23 (3.1)	1 (0.5)	2 (1.1)	7 (3.7)	13 (7)	0.001*
MACCE (3 years)	42 (5.5)	3 (1.6)	7 (4.3)	8 (4.3)	24 (12.8)	<0.001*
MACCE (7.8 years)	87 (11.6)	11 (5.8)	7 (3.7)	20 (10.8)	49 (26.5)	<0.001*
STEMI	31 (4.1)	3 (1.6)	3 (1.6)	7 (3.7)	19 (10.2)	<0.001*
Stroke	23 (3.1)	3 (1.6)	3 (1.6)	4 (2.1)	13 (7)	0.005*
Late reintervention	34 (4.6)	3 (1.6)	0	11 (5.8)	20 (10.7)	<0.001*
PCI	30 (4)	3 (1.6)	0	10 (5.3)	17 (9.1)	<0.001*
Redo-CABG	2 (0.3)	0	0	0	2 (1.1)	0.1
Pacemaker	2 (0.3)	0	0	1 (0.5)	1 (0.5)	0.56
All-cause mortality	60 (8)	8 (4.2)	9 (4.8)	15 (8)	28 (15)	<0.001*
Cardiovascular mortality	40 (5.3)	3 (1.6)	5 (2.7)	8 (4.3)	25 (13.4)	<0.001*
Non-cardiovascular mortality	19 (2.6)	5 (2.6)	4 (2.1)	7 (3.7)	3 (1.6)	0.62

* Statistically significant difference. Values are represented as mean ± standard deviation and number (%), where appropriate. CABG=coronary artery bypass grafting; MACCE=major adverse cardiac and cerebrovascular event; PCI=percutaneous coronary intervention; STEMI=ST elevation myocardial infarction

### ROC Curve Analysis

**Fig. 2:**
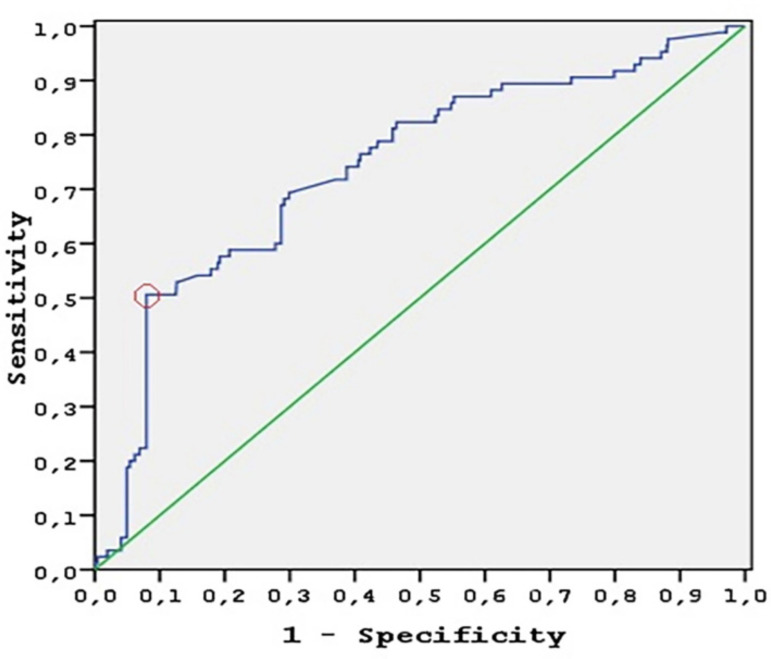
ROC curve analysis. The diagonal represents the no-effect line (AUC=0.50), with curves above this line representing increasing diagnostic accuracy. NLR accurately predicted MACCE (AUC=0.74). The circle represents the optimal cut-off to predict MACCE with a sensitivity of 50.6% and specificity of 92%. AUC=area under the curve

By ROC curve analysis, the NLR accurately predicted MACCE with an area under the receiver operating characteristics curve of 0.74 (95% CI 0.58-0.80, *P*<0.001). The positive predictive value increased with higher NLR. NLR of 4.32 was identified as the optimal cut-off to predict MACCE with a sensitivity of 50.6% and specificity of 92% (Figure 2). Based on the cut-off value, patients with high NLR (NLR>4.32) had a significantly higher MACCE rate (44.8%), compared with patients with a lower NLR (NLR≤4.32) (6.4%, HR 9.125, 95% CI 5.947-14.001, *P*<0.001).

## DISCUSSION

This study shows for the first time that NLR on admission is associated with long-term MACCE following CABG, independent of the EuroSCORE.

MACCE represents the main cause of serious morbidity and mortality following coronary revascularization and is manifested by myocardial infarction, stroke, decompensated heart failure, repeated revascularization, sudden or cardiac death. However, a standardized evaluation tool for predicting the long-term MACCE for patients undergoing coronary revascularization is lacking. Identifying a model based on demographic and clinical parameters to predict the risk of MACCE is a major concern.

NLR was found to be strongly correlated with severity of CAD^[8]^, cardiovascular event following PCI^[14,15]^, complications following CABG^[16]^ and poor midterm survival after CABG^[12]^. Although the prognostic value of NLR in cardiovascular events following PCI is well known^[2,9,14,15]^, the effect of NLR on MACCE in the CABG patients has not been adequately evaluated.

Correlated with the report of Gibson et al.^[12]^, we also found that NLR is a stronger univariable predictor of MACCE than the neutrophil or lymphocyte count. Accordingly, NLR has been reported to be the most powerful predictor of cardiovascular risk among all other parameters presented in a complete blood count in patients with CAD^[17]^. This finding may be explained by the cumulative adverse prognostic effect of neutrophilia and lymphopenia.

Similar to previous studies, our study has revealed that increased NLR is associated with factors affecting severity and complexity of atherosclerosis, such as increased age^[2,4,12,18]^, higher preoperative C-reactive protein^[10,15,18-20]^ or lower CrCl^[2,9,12,19]^ levels, increased incidence of peripheral vascular disease or insulin-dependent diabetes mellitus^[20]^ and history of smoking^[15]^.

Despite similar baseline left ventricular function, postoperative cTnI levels, or operative data like CPB time, number of distal anastomoses and endarterectomy, the higher NLR quartile shows more need for perioperative inotropic support. This finding may be related to the relationship between non-reflow phenomenon or reperfusion injury and NLR. Sen et al.^[15]^ also reported that higher NLR is associated with reperfusion injury following PCI.

As Gibson et al.^[12]^, we have found that patients with higher NLR are also more likely to require reintubation or non-invasive mechanical ventilation. Moreover, even though there is no significant difference in terms of COPD among NLR quartiles, higher NLR is related to significantly longer mechanical ventilation time. It is clearly defined that NLR elevation reflects a chronic background inflammatory state which might be exacerbated by CPB or surgery. However, this finding may also be affected by old age, which might lead to poor cardiopulmonary reserve. Unlike the previous study in CABG patients^[12]^, length of postoperative hospitalization is similar among NLR quartiles. However, length of ICU stay has found to be significantly longer in patients with higher NLR, in consequence of increased inotropic or respiratory support. Although no significant difference is detected in preoperative haemoglobin levels, chest tube drainage, or CPB time among NLR quartiles, patients with higher NLR are likely to require more blood transfusion. Although the mechanism behind this finding is unclear, we believe that the combined effect of CPB and higher NLR on systemic inflammation might cause excessive blood damage during surgery. Therefore, further studies are needed to detect the impact of increased NLR on blood transfusion in patient undergoing off-pump CABG.

Perioperative stroke is significantly more detected in patients with higher NLR. However, no difference was detected among groups in terms of transient ischemic attack or encephalopathy. The relationship between stroke and increased NLR has been shown before^[3]^; however, this relation has not been reported in patients undergoing coronary revascularization in the literature previously. Moreover, our study has also revealed the association between higher NLR and in-hospital mortality after CABG. Certainly, an increased neutrophil count is associated with increased background inflammatory state, hypercoagulability^[21]^, plaque disruption^[5,17]^ and CPB leads to neutrophil activation^[22]^. Furthermore, lymphopenia predicts a poorer outcome in patients with coronary disease^[17]^. Therefore, NLR integrates major parameters that determine MACCE, which might complicate the postoperative course. Correlated with previous publications, patients with higher NLR show a significantly lower MACCE-free survival, and also higher MACCE at 1 year, at 3 years and at 7.8 years. PCI, STEMI and stroke are detected significantly more in higher NLR quartile. As the non-cardiovascular mortality is not to be associated with higher NLR, unlike cardiovascular mortality, the higher all-cause mortality seems to be mainly affected by cardiovascular mortality.

Despite NLR can be obtained simply in a routine blood test, to date, there is no consensus about an NLR cut-off value. Predicting in-hospital mortality following PCI for STEMI, an NLR of 5.44 and 5.9 were reported as cut-off values^[2,23]^. Accordingly, an NLR of >5.25 was found as the cut-off to predict short-term mortality in patients with peripheral arterial disease who presented with CLI^[24]^. Moreover, Kordzadeh et al.^[25]^ revealed an NLR of 5 as a cut-off to predict 30-day morbidity in ruptured abdominal aortic aneurysm. To our knowledge, in this study, we have identified for the first time that an NLR of 4.32 is the optimal cut-off to predict MACCE following elective isolated CABG.

In addition, NLR has found to be the only predictor of MACCE in low-risk, medium-risk and in high-risk EuroSCORE groups. Thus, its long-term prognostic value is independent of surgical risk factors. It seems to be used as an MACCE predictive index in the follow-up for every patient.

The primary limitations of the present study are the reflection of a single centre experience and retrospective design. Moreover, the use of preoperative blood result, rather than repeated samples at regular intervals, does not allow assessment of the change in NLR over time. However, our population contains homogeneous, consecutive unselected CABG patients, relevant to most patients undergoing CABG in the general population. Moreover, all patients were submitted to ON-BH CABG under the same experienced surgeon supervision; therefore, the factors which interact with the frequency of MACCE due to differences in surgical technique were excluded. Furthermore, the single centre nature also ensured that all the blood samples were studied with the same haematology analyser, which ensured the potential variation.

## CONCLUSION

NLR is independently associated with long-term MACCE in patients undergoing CABG. This simple, inexpensive predictor might help identify individuals at risk for adverse outcome who might be potential candidates for a more aggressive therapeutic approach to control risk factors. NLR should be used to single out patients at increased risk of MACCE. Future prospective studies and large-scale randomized controlled trials are needed to establish standardized cut-off values for NLR predicting MACCE following CABG and to clarify the underlying mechanisms.

**Table:** 

Author's roles & responsibilities	
OG	Substantial contributions to the conception or design of the work; or the acquisition, analysis, or interpretation of data for the work; drafting the work or revising it critically for important intellectual content; final approval of the version to be published
GK	Substantial contributions to the conception or design of the work; or the acquisition, analysis, or interpretation of data for the work; agreement to be accountable for all aspects of the work in ensuring that questions related to the accuracy or integrity of any part of the work are appropriately investigated and resolved
HO	Substantial contributions to the conception or design of the work; or the acquisition, analysis, or interpretation of data for the work; agreement to be accountable for all aspects of the work in ensuring that questions related to the accuracy or integrity of any part of the work are appropriately investigated and resolved
IHK	Substantial contributions to the conception or design of the work; or the acquisition, analysis, or interpretation of data for the work; agreement to be accountable for all aspects of the work in ensuring that questions related to the accuracy or integrity of any part of the work are appropriately investigated and resolved
YV	Drafting the work or revising it critically for important intellectual content; final approval of the version to be published
AE	Substantial contributions to the conception or design of the work; or the acquisition, analysis, or interpretation of data for the work; drafting the work or revising it critically for important intellectual content
AY	Drafting the work or revising it critically for important intellectual content
SE	Drafting the work or revising it critically for important intellectual content
